# Understanding Drivers of Loss to Follow‐Up in HIV Care in Oromia Regional State, Ethiopia: A Qualitative Grounded Theory

**DOI:** 10.1002/hsr2.73433

**Published:** 2026-10-26

**Authors:** Fikru Tafese, Matebu Gezahegn, Zeriun Kura, Eshetu Alemayehu, Yadeta Ayana, Chala Bafikadu, Gusha Belako, Tesfaye Zerfu, Birhanu Kenate Sori, Yadeta Dassie, Firaol Bekele, Negash Sime, Tizta Tilahun Degfie, Daniel Yilma, Yohannes Ejigu, Yohannes Kebede, Mirkuzie Woldie

**Affiliations:** ^1^ Department of Health Policy and Management Jimma University Ethiopia; ^2^ Department of Epidemiology Jimma University Ethiopia; ^3^ Oromia Health Bureau Ethiopia; ^4^ Haramaya University College of Health and Medical Sciences Ethiopia; ^5^ Department of Reproductive Health and Population Studies Bahir Dar University Ethiopia; ^6^ Department of Internal Medicine Jimma University Ethiopia; ^7^ Department of Health, Behavior and Society Jimma University Ethiopia; ^8^ Fenot Associates Addis Ababa Ethiopia

**Keywords:** community engagement, Ethiopia, loss to follow‐up (LTFU), Oromia, structural factors, tailored interventions

## Abstract

**Background:**

Despite significant advancements in antiretroviral therapy (ART), loss to follow‐up (LTFU) remains a critical challenge in HIV care, particularly in low‐resource settings like Ethiopia. LTFU contributes to increased morbidity and mortality rates, heightened transmission risks, and treatment failure. While individual and clinical factors have been widely studied, structural drivers of LTFU are less understood. Hence, this study aimed to understand the structural factors driving LTFU in HIV care.

**Methods:**

A qualitative study using grounded theory was conducted. Data were collected through Key Informant Interviews (KIIs) with program staff, Focus Group Discussions (FGDs) with social support groups, and group interviews with program coordinators and regional directors. Thematic analysis was performed using ATLAS. ti. Software version 7.5.18, and findings were reported following the Standards for Reporting Qualitative Research (SRQR).

**Results:**

Four key themes emerged from the analysis: (1) programmatic and service challenges, including drug supply interruptions, inadequate service integration, and human resource shortages; (2) weak transfer, linkage, and tracing systems, marked by gaps in referral networks and untraceable client identifiers; (3) cultural and socio‐behavioral factors, such as stigma, discrimination, and reliance on faith‐based healing practices. Participant quotations highlighted the pervasive impact of stigma and weak referral systems on patient disengagement from HIV care; and (4) integration gaps in social change and stakeholder engagement, including weak counseling services and limited collaboration with non‐governmental organizations and community organizations.

**Conclusion:**

The findings underscore the critical role of structural factors in shaping retention in HIV care in Oromia. Addressing these barriers may benefit from a multi‐faceted approach, including strengthening health systems, improving referral and tracing mechanisms, reducing stigma, and enhancing stakeholder engagement. These insights provide a foundation for targeted interventions to reduce LTFU and improve HIV care outcomes in the region.

**Abbreviations**
AIDS: acquired immunodeficiency syndrome
ART: antiretroviral therapy
EPHI: Ethiopian public health institute
FGD: focus group discussion
HIV: human immunodeficiency virus
KII: key informant interview
LTFU: loss to follow‐up
ORHB: Oromia regional health bureau
PLHIV: people living with HIV
UNAIDS: Joint United Nations Program on HIV/AIDS

## 1 Introduction

Over the past four decades, significant advancements in antiretroviral therapy (ART) have dramatically reduced the global and local burden of HIV [1, 2, 3]. These developments have not only lowered infection rates but also improved the longevity and quality of life for individuals living with HIV, enabling many to live long, healthy lives comparable to those without the virus [4, 5]. The accessibility of ART and ongoing public health initiatives have been instrumental in this transformative journey [6, 7, 8, 9, 10].

Despite these successes, retaining HIV patients in lifelong care remains a significant challenge, primarily due to loss to follow‐up (LTFU) [11, 12, 13]. LTFU in the context of HIV care refers to patients who stop coming for treatment within a specified period. This study adopted the recent definition of the World Health Organization (WHO), which defines “lost to follow‐up” as patients who have not been seen at the facility or community service delivery site for 28 days or more since their last missed appointment. This includes missed antiretroviral drug refills in a facility or community setting 14]. At the actual program level, this definition is adopted nationally to classify patients as LTFU.

Despite Ethiopia's significant strides in expanding HIV care and treatment services, LTFU remains a major challenge [1, 2, 15], particularly in regions like Oromia, where facility reports indicate alarming rates of disengagement from care. LTFU poses a considerable threat to global and local targets aimed at ending AIDS as a public health problem, undermining the progress achieved in HIV treatment and control [14, 15, 16, 17, 18, 19, 20]. It is a multifaceted issue [1, 21] influenced by individual, interpersonal, community, and health system factors, and it remains a critical barrier to achieving the UNAIDS 95‐95‐95 targets [20, 21, 22, 23]. These multifaceted and interrelated factors can be understood by structurally underpinning them [24]. Structural factors are systemic and contextual determinants that indirectly influence health outcomes by shaping the environment in which healthcare services are delivered and accessed [24, 25, 26]. These factors operate at the macro level and include elements such as health system policies, service delivery models, resource availability, social norms, stigma, and community engagement [27, 28, 29, 30]. In the context of HIV care, structural factors can affect retention in care and adherence to ART by influencing access to services, the quality of care, and the social support available to individuals living with HIV [27, 28, 29, 30]. Structural factors encompass programmatic and service factors, transfer, linkage, and tracing systems, cultural and behavioral factors, and integration within social change and stakeholder engagement [27, 28, 29, 30]. In Ethiopia, a recent meta‐analysis revealed a pooled retention rate among HIV patients of 70.65%. The overall LTFU rates were 15.17%, transfer‐outs 11.17%, and deaths 6.75% [31]. Significant attrition factors were identified, including being unmarried, non‐disclosure of HIV status, poor drug adherence, and poor health status [31]. Factors facilitating retention included the absence of opportunistic infections, normal hemoglobin levels, and non‐substance use [31].

Most of the current evidence on LTFU has primarily focused on quantitative estimation of its burden and identification of individual socio‐demographic and clinical factors as contributors to LTFU, while a broader view of the structural underlying drivers has remained unexplored. Hence, understanding the structural factors is critical for uncovering the root causes and addressing the challenges of retention in HIV care and reducing LTFU in the context of Oromia, Ethiopia.

## 2 Methods and Materials

### 2.1 Study Area and Period

The study was conducted in Oromia Regional State, Ethiopia's largest and most diverse region, with a population of about 40 million and an area of 359,619.8 square kilometers [32]. Oromia is administratively divided into 21 zones, 19 towns, 336 districts, and 7021 kebeles (villages) [33]. The region has an extensive health infrastructure, including 104 hospitals, 1405 health centers, 7090 health posts, and 430 ART‐providing sites. In 2022, an estimated 610,000 people were living with HIV in Ethiopia, with about 25% of them residing in Oromia [34].

HIV service delivery is organized across multiple levels: regional health bureaus coordinate programs, zonal and district offices manage facilities, and health centers and hospitals provide ART initiation, follow‐up, and counseling. Health posts and community structures support linkage, tracing, and adherence. Communities are highly diverse, rural, urban, agrarian, and pastoralist, reflecting varied sociocultural contexts in which HIV services are accessed. HIV services are provided free of charge in Ethiopia, though other health services (e.g., laboratory tests, imaging, non‐HIV care) may require payment.

Oromia regional state is particularly relevant for studying structural drivers of LTFU because it combines a large HIV‐affected population, wide geographic and cultural diversity, and a multi‐layered health system with both strengths (broad ART coverage, free ART provision) and weaknesses (fragmented referral systems, resource constraints, and sociocultural barriers). These features make Oromia an ideal setting for examining how structural drivers influence retention in HIV care. The study was conducted between May 5 and June 5, 2022.

### 2.2 Qualitative Approach and Research Paradigm

This study employed a qualitative research design, grounded in an abbreviated version of grounded theory (AGT) [35], emphasizing iterative data collection, constant comparative analysis, and theoretical saturation, to understand structural factors underlying LTFU in HIV care. Situated within the constructivist paradigm [35], the research emphasizes understanding the normative meanings and experiences of participants, aligning with its goal of uncovering the underlying barriers and challenges faced by individuals in HIV care programs. A grounded theory research design was chosen as it facilitates the inductive development of key concepts and dimensions emerging directly from systematically gathered and analyzed data, providing a rigorous framework for understanding the underlying structural drivers of LTFU. The study utilized the AGT design, which focused on core elements such as open coding, categorization, and theory development, balancing depth of analysis with practical constraints from the iterative process (constant comparison and theoretical sampling).

### 2.3 Study Population and Participants

The study purposively included diverse groups at the system, facility, and community levels to capture perspectives on HIV care and the structural drivers of loss to follow‐up in HIV care (Table 1).

**Table 1:** Study participants and sample size matrix.

Data source type	Participant type and composition	Site(s)	Total number of sources
Key informant interviews (KIIs)	LTFU ART patients (Jimma = 1, Guji = 1)	Jimma, Guji	2
	Never LTFU ART patients (Adama = 1, Guji = 1)	Adama, Guji	2
	HIV association leaders (Adama = 1, Jimma = 1)	Adama, Jimma	2
	Peer ART supporters (Jimma = 1, Guji = 1)	Jimma, Guji	2
Focus group discussions (FGDs)	Social supports: religious leaders, opinion leaders, Edir leaders	Jimma, Guji	2 FGDs (8–10 participants each)
Group interviews	Facility‐level: care providers, data managers, ART dispenser pharmacists, ART clinic focal persons	Adama, Guji	2
	Program coordinators: town HIV/ART focal (2), zonal HIV focal (2)	Adama, Jimma	2
	Regional level: ORHB HIV/AIDS directors and coordinators (2), ORHB CDC (1), ORHB ICAP (1)	ORHB	1
Overall total data sources			15

### 2.4 Data Collection Tools and Procedures

The study employed a combination of qualitative data collection methods to gather comprehensive insights from a diverse range of stakeholders. These methods included Focus Group Discussions (FGDs), Key Informant Interviews (KIIs), and Group Interviews, each tailored to capture the perspectives of different participant groups.

1. Focus group discussions (FGDs):

Two FGDs were conducted with social support groups, which included religious leaders, opinion leaders, and Edir leaders (An “Edir” is a traditional voluntary community organization established to provide mutual support during social challenges, particularly in managing funeral arrangements and offering emotional and financial assistance to bereaved families, reflecting a deep‐rooted culture of solidarity and collective responsibility [36]. These discussions aimed to explore the community‐level dynamics, cultural influences, and social support mechanisms that impact retention in HIV care.

2. Key informant interviews (KIIs):

Key informant interviews were conducted with eight participants: two PLHIV with no history of loss to follow‐up (LTFU), two PLHIV who had experienced repeated instances of LTFU, two adherence supporters who have long years of experience in peer support, and two HIV association leaders. These interviews provided in‐depth, individualized perspectives on the factors influencing retention and discontinuation in HIV care.

3. Group interviews:

Five Group interviews were conducted with five distinct groups: two involving care providers, two with program coordinators at the zonal/town level, and one with regional‐level program directors. These sessions aimed to gather insights from healthcare professionals and program managers on systemic challenges, operational barriers, and strategies to improve retention in HIV care.

Participants were purposively selected through consultation with Oromia Regional Health Bureau HIV program coordinators and technical consultants. This process ensured that individuals most relevant to HIV service delivery and patients' experiences were included. The purposive approach was chosen to gain depth of information from participants whose roles, responsibilities, and lived experiences positioned them to provide critical insights into structural drivers of retention and loss to follow‐up (LTFU).

#### 2.4.1 Justification for Purposive Selection

We sought to gain depth of data by purposively selecting participants whose lived experiences, program management responsibilities, professional expertise, and social roles positioned them to provide rich and diverse insights. ART program managers, providers, pharmacists, PLHIV, and support groups were deliberately included because of their direct involvement in service delivery, policy oversight, and lived experience with HIV care. Their engagement ensured that perspectives from both the health system and the community were represented, allowing us to capture systemic facilitators and barriers across the continuum of care. This purposive selection was therefore justified by the relevance of participants' roles and experiences in illuminating structural drivers of retention and disengagement.

#### 2.4.2 Focus Group Discussions (FGDs)

Two FGDs were conducted, each with 8–10 participants. These comprised community influencers such as religious leaders, Edir leaders, and opinion leaders, selected to represent community perspectives shaping adherence, tracing, and support.

- Sampling rationale: To explore contrasting experiences, we included both PLHIV with repeated LTFU episodes and those consistently retained in care. This deliberate balance enabled comparison of barriers and facilitators of retention. Pediatric and adolescent clients were represented directly or through caregivers to capture age‐specific challenges.
- Identification of LTFU patients and those with no episodes of LTFU: Participants were identified through ART clinic records and referrals from peer supporters/adherence counselors. This approach ensured the inclusion of individuals with firsthand experiences of disengagement while maintaining confidentiality and ethical safeguards. For patients with a history of LTFU, adherence managers who routinely monitor attendance and follow‐up reviewed facility registers to confirm prior episodes of disengagement.

### 2.5 Data Analysis and Trustworthiness

The data analysis was conducted using open coding with ATLAS.ti software version 7.5.18. Open coding: Initial themes (e.g., “drug shortages,” “stigma,” “tracing challenges”).

Axial coding: Connections between themes (e.g., supply chain gaps → appointment scheduling → LTFU). Selective coding: Core category identified: Structural–implementation disconnect in HIV care.

The process involved a contextual interpretation of the texts while preserving their original meanings. A draft codebook was developed through multiple readings of the initial rich transcripts and refined through iterative discussions among the research team. Three independent coders conducted the coding process to ensure consistency and reduce bias. Following coding, thematic analysis was performed to categorize codes and identify emergent themes and their properties. The themes related to factors contributing to LTFU from HIV care were described conceptually and supported by relevant quotations from participants.

#### 2.5.1 Trustworthiness of the Study

To ensure the rigor and trustworthiness of the study, the four components of qualitative research: credibility, dependability, transferability, and conformability [35] were addressed as follows, and the standards of reporting for qualitative research (SQRL) were used to report this qualitative research [37].

#### 2.5.2 Credibility

Credibility was ensured through prolonged engagement with the study context, triangulation of data sources (KIIs, GIs, and FGDs), and member checking. Participants were provided with summaries of their responses to confirm accuracy and interpretation. Additionally, the use of multiple coders and iterative discussions during codebook development enhanced the credibility of the findings.

#### 2.5.3 Dependability

Dependability was achieved by maintaining a detailed audit trail of the research process, including raw data, coding decisions, and thematic development. The use of ATLAS.ti software ensured systematic and transparent data management. Furthermore, the involvement of three independent coders and regular team discussions minimized inconsistencies in data interpretation.

#### 2.5.4 Transferability

To enhance transferability, thick descriptions of the study context, participant characteristics, and data collection procedures were provided. This allows readers to assess the applicability of the findings to other settings. The inclusion of diverse participant groups (e.g., healthcare providers, patients, and community leaders) also contributed to the richness of the data.

#### 2.5.5 Conformability

Conformability was ensured by maintaining objectivity and minimizing researcher bias. The use of independent coders, an audit trail, and reflexive practices (e.g., documenting researcher assumptions and biases) helped ensure that the findings were grounded in the data rather than researcher perspectives.

#### 2.5.6 Reflexivity

The principal investigator (PI), leveraging experience in HIV program coordination and health systems, focused the study while avoiding bias. A qualitative research expert ensured methodological rigor and balanced interpretation. Trained data collectors, supervised closely, and built trust with participants while remaining neutral. The team emphasized credibility through continuous reflection, collaboration, and supervision, ensuring authentic representation of those affected by LTFU.

## 3 Results

### 3.1 Study Population and Participant' Characteristics

The study drew data from 15 sources through key informant interviews, focus group discussions, and group interviews. Distinct participant groups contributed, reflecting diverse roles and perspectives across the HIV care continuum. These included PLHIV with varied care trajectories (repeated LTFU vs. consistently retained), facility‐based providers (clinicians, pharmacists, pediatric specialists, data managers), program managers and coordinators at zonal, town, and regional levels, and community stakeholders such as religious leaders, Edir leaders, opinion leaders, peer supporters, and HIV association leaders (Table 2).

**Table 2:** Participant characteristics.

Data collection modality	Participant category/stakeholder level	Sites	Number of sources	Total participants
Key informant interviews (KIIs)	Lost to follow‐up (LTFU) ART patients. Never LTFU ART patients, HIV association leaders, peer ART supporters	Adama, Jimma, Guji	8	8 (1 per session)
Focus group discussions (FGDs)—community	Social support (religious, opinion, and Edir leaders)	Jimma, Guji	2	16–20 (8–10 per session)
Group interviews (GIs)— facility	Health service providers (data managers, ART dispenser pharmacists, ART clinic focal)	Adama, Guji	2	
Group interviews (GIs)—zone health departments and town health office	Zonal HIV program focal and town health office HIV program focal	Adama, Jimma	2	4 (2 per session)
Group interviews (GIs)— regional	HIV/AIDS program director, care and treatment team coordinators, and technical advisors	Oromia Regional Health Bureau	1	4

This study identified four key themes contributing to loss to follow‐up (LTFU) in HIV care, including programmatic and structural challenges, weak transfer, linkage, and tracing systems, cultural and behavioral factors, and stakeholder engagement and integration (summarized in Table 3). Each theme reflects systemic, operational, and sociocultural barriers that impact retention in HIV care.

1. Programmatic and structural challenges include drug stockouts, inadequate clinic design, and gaps in human resources, all of which disrupt service delivery and compromise confidentiality.
2. Weak transfer, linkage, and tracing systems highlight inefficiencies in referral networks and client tracking due to incorrect data and logistical barriers.
3. Cultural and behavioral factors reveal stigma, discrimination, and the influence of faith‐based institutions on treatment adherence.
4. Stakeholder engagement and integration underscore weak social and behavior change (SBC) strategies and limited collaboration among stakeholders, hindering community‐level support.

**Table 3:** Summary of identified key themes and subthemes along with their main descriptions.

Theme	Description
1. Programmatic and structural challenges	Drug stockouts, poor clinic design, and human resource gaps.
2. Weak transfer, linkage, and tracing systems	Inefficient referrals and tracing due to incorrect data.
3. Cultural and behavioral factors	Stigma, faith‐based institutions influence
4. Stakeholder engagement and integration	Weak SBC strategies and low stakeholder engagement

Below are each theme and sub‐themes supported by participant quotations and subthemes, to elucidate the multifaceted challenges in sustaining HIV care continuity.

### 3.2 Theme 1: Programmatic and Structural Challenges in ART Service Delivery

Three interrelated sub‐themes emerged under this theme: drug supply and accessibility issues, service delivery and integration gaps, and human resource shortages. Together, they highlight systemic weaknesses that undermine continuity of HIV care.

#### 3.2.1 Sub‐Theme 1: Drug Supply and Accessibility Issues

Frequent Antiretroviral Therapy (ART) stockouts severely disrupted treatment schedules and forced healthcare providers to alter standard protocols, heavily impacting patient retention. Participants highlighted a critical inconsistency in the supply chain, noting that “sometimes we face stockouts to zero level” (FGD, ART dispensing pharmacist). This problem was particularly acute for younger patients. As one pharmacist explained, “the supply of specific pediatric drugs from top to down is not consistent… As a result, we were breaking down the adult dose (Abacavir) and giving it to children” (EGD, ART dispensing pharmacist). To cope with these systemic shortages, which included a lack of “Dolutegravir loose form” and “3rd line drugs,” facilities routinely had to “shift the appointment date to solve this problem” (EGD, ART dispensing pharmacist). Ultimately, healthcare workers viewed these national supply chain failures as a direct driver of patient drop‐out, concluding that the rigid supply system “has its own contribution to LTFU (loss to follow‐up)” (EGD, pharmacy).

#### 3.2.2 Sub‐Theme 2: Service Delivery and Integration Gaps

Clinic layouts often exposed clients in waiting areas, eroding their sense of safety and dignity. Confidentiality was also inadequately safeguarded, with sensitive information vulnerable to disclosure. Together, these weaknesses reinforced stigma and discouraged retention in care.

The setups of our ART clinic expose HIV clients … not in a way that keeps confidentiality.

(FGD, focal person)

This distinction is critical: privacy refers to the physical environment (e.g., clinic layout exposing clients), while confidentiality refers to the protection of client information. In practice, both were compromised, undermining trust in ART services.

#### 3.2.3 Sub‐Theme 3: Human Resource Shortages

Human resource constraints emerged as a critical barrier to the quality and sustainability of ART service delivery. Two interlinked dimensions were highlighted: staff turnover and inadequate training capacity, and compromised professional standards in service provision.

High staff turnover and limited training capacity: Frequent staff turnover weakened program continuity, while access to training for new personnel was restricted. Training of trainers (ToT), once coordinated by the Ministry of Health and regional government, has been narrowed to universities and hospitals, excluding frontline program staff. This shift undermined the ability of facilities to provide in‐service training and fill urgent gaps in human resources.

Previously the minister of health and regional government were giving training of trainers as a resource person for people on the program. However, currently it is very specific and mentorship is given by hospitals or from university. They are not including people on the program in taking training of trainers. As a result, we are unable to even provide in‐service training in areas where there is a shortage of people.

(FGD, zonal focal person)

This lack of systematic training pipelines left facilities dependent on ad‐hoc mentorship, often requiring staff to seek orientation from universities rather than receiving structured capacity‐building within the health system. The absence of training of trainers (ToT) mechanisms limited the ability to scale up skilled providers, exacerbating shortages during turnover.

Compromised professional standards in service provision: Human resource gaps also translated into compromised professional standards. In several facilities, ART drugs were dispensed by non‐pharmacists due to shortages of trained personnel, raising concerns about service quality and patient safety.

ART drugs should be dispensed by pharmacists, but in our area, they are dispensed by other providers.

(FGD, focal person)

This practice not only undermined professional norms but also risked eroding patient trust in ART services, particularly in contexts where confidentiality and technical accuracy are paramount.

Across the three sub‐themes (Drug Supply and Accessibility Issues, Service Delivery and Integration Gaps, and Human Resource Shortages), a consistent picture emerges of systemic weaknesses that undermine continuity of HIV care. Unreliable drug supply, inadequate clinic environments, and insufficient human resources interact to create a fragile system where patients face repeated disruptions, diminished trust, and reduced quality of care, which may undermine retention and long‐term outcomes. Building on these structural challenges. Theme 2 examines weaknesses in transfer, linkage, and tracing systems, focusing on gaps in referral processes and patient follow‐up mechanisms.

### 3.3 Theme 2: Weak Transfer, Linkage, and Tracing Systems With Sub‐Themes: Transfer and Referral System Gaps and Patient Tracing

These themes describe challenges in referral and client tracking that affect retention in care, as well as gaps in transfer and referral systems and tracing mechanisms.

Transfer and referral system gaps: challenges in transferring clients and limited options for obtaining medication.

If possible, the solution is an emergency request. If the emergency is not successful, borrowing from other health facilities.

(EGD, zonal focal)

Patient tracing: difficulties in locating and contacting clients due to incorrect phone numbers and clients seeking care at new facilities.

Even though I haven't known the reason, most of the time their phone number is not the correct one.

(KII, peer supporter)

Digital disconnect was also a key finding under this theme; the lack of interoperable systems hindered self‐transfer. A returned client noted: “I migrated for work… couldn't access ART until I returned.”(KII, case)

Taken together, the gaps in transfer, referral, and tracing systems reveal how structural disconnects weaken continuity of HIV care. Limited referral options, inaccurate contact information, and non‐interoperable digital systems leave mobile and returning clients vulnerable to disengagement. These systemic weaknesses were perceived to disrupt treatment and expose patients to broader social pressures. Building on these findings, Theme 3 turns to cultural and socio‐behavioral factors, where stigma, community narratives, and faith‐based influences were perceived to shape retention and adherence outcomes.

### 3.4 Theme 3: Cultural and Socio‐Behavioral Factors

Two major sub‐themes emerged: Stigma and Discrimination and the Role of Faith‐Based Institutions. Together, they illustrate how cultural beliefs and social dynamics shape retention in HIV care.

#### 3.4.1 Sub‐Theme 1: Stigma and Discrimination

Stigma operated at multiple levels, creating barriers to care and undermining adherence:

Individual level: Clients feared disclosure within families and close networks, leading to secrecy and disengagement.

Despite what you tell the community about HIV/AIDS, I do not think they will hear or accept your message. They prefer to stigmatize you than support you.

(KII, case)

Community level: Social norms and gossip reinforced discrimination, discouraging clients from attending ART clinics. Religious and cultural narratives often framed HIV as a moral failing, intensifying shame.

Facility level: Clinic environments compromised confidentiality and privacy, reinforcing stigma. Clients reported that being visibly identified as HIV‐positive in waiting areas discouraged attendance.

The setups of our ART clinic expose HIV clients to other individuals … not in a way that keeps confidentiality.

(FGD, focal person)

These responses imply that stigma is a layered phenomenon: personal fear, community judgment, and institutional shortcomings intersect to perpetuate disengagement.

#### 3.4.2 Sub‐Theme 2: Role of Faith‐Based Institutions

Faith‐based institutions emerged as both barriers and facilitators, depending on interpretation and practice. Faith‐based institutions were referred to frequently across FGDs and KIIs. They functioned as a dual influencer, a source of resilience and support for some, but a barrier for others when messages conflicted with biomedical care. Their role was therefore analytically positioned as both facilitator and barrier, highlighting the need for structured engagement with religious leaders in HIV programs.

Facilitators: Religious practices provided spiritual comfort and psychosocial support. Some clients combined ART with faith rituals, demonstrating coexistence rather than conflict.

In addition to using holy water, I did not discontinue my medication … I was using both ART drugs and holy water at the same time.

(KII, case)

Barrier: In other cases, leaders discouraged ART adherence, framing it as a lack of faith. This misinformation led some clients to interrupt treatment.

Some believe that taking medication signifies a loss of trust in God … unfortunately, religious leaders sometimes spread misinformation, leading individuals to stop ART treatment.

(FGD, religious leader)

The cultural and socio‐behavioral factors outlined in this theme, particularly stigma, discrimination, and the dual role of faith‐based institutions are perceived as how social narratives and community dynamics may shape retention in HIV care. Yet these influences do not operate in isolation; they intersect with programmatic strategies and stakeholder actions. Building on this, Theme 4 examines the integration of ART with community and stakeholder engagement, highlighting weak social and behavior change (SBC) strategies and limited collaboration with stakeholders as critical gaps that further undermine continuity of care.

### 3.5 Theme 4: Integration of ART With Community and Stakeholder Engagement With Sub‐Themes: Weak Social and Behavior Change (SBC) Strategies and Stakeholder Engagement and Integration

This theme highlights the impact of weak implementation of SBC strategies and limited stakeholder engagement. Socio‐economic and demographic segmentation is critical in understanding disparities in HIV care engagement. Specific populations, such as pastoralists, rural residents, and those in insecure settings, face unique challenges. As noted by the ART provider,

“The other problem related to LTFU is psychosocial factors… Children usually challenge us due to psychosocial‐related factors… HIV‐positive children do not change our counseling into practice; it is a great challenge.” (EGD, ART provider) A case participant suggested a potential solution, stating, “It is possible to go to a nearby health facility… Then, linking the two health facilities via phone call to obtain clear information about the patient… This is a good solution.”

(KII, case)

Weak social and behavior change (SBC) strategies: Ineffective social supports, counseling, and the decline in health education initiatives were identified as challenges.

HIV‐positive children do not change our counseling into practice; it is a great challenge.

(EGD, ART provider)

Limited support from organizations, as well as decreased engagement from community leaders. Despite attempts to engage faith‐based institutions, their full potential remains untapped, highlighting the need for stronger collaboration and integration.

In both Muslim and Christian teachings, preserving human life from harm, poisoning, and diseases is paramount. However, due to a lack of awareness, some believe that taking medication signifies a loss of trust in God, and that faith alone can heal. Unfortunately, religious leaders sometimes spread misinformation, leading individuals to stop their ART treatment. We know of cases where people have interrupted their medication because of this.

(FGD, religious leader, p5)

The implementation quality and reach of social and behavior change activities, such as counseling and education services within ART facilities, play a crucial role in shaping the stigma associated with HIV, as well as the level of social support and community awareness about HIV‐positive individuals or those on ART. Despite positive reports about provider‐client relationships, significant limitations exist in educational and communication services, resulting in persistent stigma, discrimination, and inadequate emotional resilience and support for those affected. A key informant noted,

“Health workers from health centers also create awareness and motivate people to be tested for HIV… They advise us that ART should be continuously taken for life long.” (KII, control) However, another participant highlighted the lack of community engagement, stating, “Before a few years ago, health education was given to the community. But currently, I think it has stopped… The communities think this disease is eradicated, even though this disease is currently spreading more than the previous times.

(FGD, religious leader)

Another main social factor was found to be a lack of adequate food, which was raised as even a prerequisite to take their daily ART drugs, for example, one participant stated this as;

The main reason for LTFU is that the medication needs a diet. One has to eat to take ART drugs. However, HIV clients face a shortage of food/diet and decide to LTFU. For instance, I sometimes swallow the drug on an empty stomach without eating food. When I swallow the drugs without eating, it has a burning sensation. Without eating food, the drugs damage the clients. Swallowing drugs by itself may make you hungry. There may be clients who are unable to eat their breakfast, lunch, and dinner. There may also be those who have many family members. They may not get sufficient food to eat. Due to this, they decide to stop taking the drugs and LTFU.

(KII, case)

Stakeholder engagement and integration: Effective stakeholder and social engagement, along with service integration, is crucial for HIV and ART services. Multiple stakeholders must be involved in the continuum of care, from screening to lifelong adherence to ART. Essential services include educational and counseling support, social institutions that foster emotional resilience, and NGOs that provide various forms of assistance. A peer supporter noted,

“Those people from the mining area go to this foundation to get a rest… Mother Teresa supports those who have TB, HIV, and orphans… They also have adherence supporters.” (KII, peer supporter) However, another participant highlighted the lack of support, stating, “There is no one who supports/encourages people to be tested for HIV/AIDS… Unless HIV clients go to ART centers by themselves, no one supports them to visit ART sites.”

(KII, control)

As stated by a peer supporter, there are also occasions of collaborating with health extension workers to support patients to follow up on their HIV care: “We collaborate with health extension workers”(KII, peer supporter).

The results show that structural inefficiencies, weak referral and tracing systems, socio‐behavioral barriers, and limited stakeholder engagement are collectively perceived as important contributors to loss to follow‐up in HIV care.

To move from evidence to action, these challenges are distilled into a practical framework that links barriers with targeted solutions. Table 4 presents this framework, highlighting priority interventions to strengthen ART service delivery, improve continuity of care, reduce stigma, and enhance stakeholder collaboration in Oromia.

**Table 4:** Framework to reduce LTFU in HIV care in Oromia regional state, Ethiopia.

Challenges and factors	Key areas	Solutions/actions
**1. Programmatic and structural challenges in ART services delivery**		
Drug supply	Strengthening the supply chain	Emergency reserve for shortages
Services delivery	Redesign clinics for confidentiality	Integrate services for efficiency
HR challenges	Provide training and mentorship	Recruit and retain skilled staff
**2. Weak transfer, linkage, and tracing system**		
Transfer gaps	Standardize the referral system	Provide transportation support
Tracing issues	Develop a digital tracking system	Train peer supporters and HEWs
**3. Cultural and behavioral factors**		
Stigma	Awareness campaigns	Promote PLHIV ambassadors and psychosocial support
Faith‐based influences	Engage religious leaders	Use culturally sensitive messages
Community support	Strengthen the support group	Initiate economic empowerment programs
**4. Implementation gap in social change and stakeholders' engagement**		
Weak SBC strategies	Revitalize health education	Effective counseling
Stakeholder engagement	Regional taskforce	Capacity building initiatives
Opportunities for improvement	Scale up peer support	Pilot innovative tracing models and evaluate the impact
Monitoring and evaluation	Indicators: data collection: reporting and feedback	Retention rates, adherence rate, stigma reduction ratessurveys, focus groups, and routine dataregular progress report

## 4 Discussion

This study identified four interrelated themes contributing to loss to follow‐up (LTFU) in HIV care: programmatic and structural challenges, weak transfer, linkage, and tracing systems, cultural and socio‐behavioral factors, and stakeholder engagement and integration. Together, these findings highlight how systemic inefficiencies, sociocultural dynamics, and limited stakeholder collaboration are perceived to undermine retention in HIV care in Oromia.

### 4.1 Programmatic and Structural Challenges

Frequent drug stockouts, inadequate clinic design, and human resource shortages disrupted continuity of care. These findings align with prior studies in sub‐Saharan Africa that link supply chain weaknesses and staff turnover to poor ART adherence [7]. Similarly, in Kenya, inadequate integration of HIV services with other healthcare services compromised patient confidentiality and discouraged retention [38]. Our analysis extends this by distinguishing privacy (physical exposure in clinic layouts) from confidentiality (protection of client information). Both were compromised, eroding trust in services. This suggests that retention strategies must go beyond drug availability to include facility redesign for privacy and training for confidentiality safeguards.

### 4.2 Weak Transfer, Linkage, and Tracing Systems

Significant gaps in referral networks and tracing mechanisms were observed, echoing findings from South Africa where weak referral systems and poor communication between facilities led to high rates of LTFU [39]. In Malawi, incorrect patient contact information and limited tracing capacity were identified as major barriers [32]. Our study highlights the dual challenge of inaccurate contact information and patients restarting care at new facilities, complicating tracing efforts. This underscores the need for standardized patient identification systems and data‐sharing mechanisms across facilities. Leveraging community health workers and peer supporters to enhance tracing and linkage also emerged as a promising strategy, consistent with evidence from other settings [33].

### 4.3 Cultural and Socio‐Behavioral Factors

Stigma, discrimination, and reliance on faith‐based healing practices were significant barriers to ART adherence. These findings mirror studies in Addis Ababa, where stigma and fear of disclosure were major reasons for LTFU [34], and in Nigeria, where cultural beliefs and traditional healing competed with biomedical HIV care [40]. Our study adds depth by showing stigma at individual, community, and facility levels, and by highlighting the digital disconnect faced by migrant clients unable to self‐transfer due to non‐interoperable systems. Faith‐based institutions emerged as a dual influence: for some, religious practices facilitated adherence by providing spiritual support; for others, misinformation discouraged ART use. This complexity underscores the need for culturally sensitive interventions that engage faith leaders as partners in adherence promotion.

### 4.4 Stakeholder Engagement and Integration

Weak implementation of SBC strategies and limited stakeholder collaboration hindered retention. Similar findings have been reported in Tanzania, where the lack of community engagement undermined HIV care [41]. Our study highlights the potential of peer support groups and health extension workers to bridge these gaps, consistent with evidence from Mozambique where community‐based peer support improved retention [42]. However, NGOs and community organizations remain underutilized, pointing to the need for stronger multi‐sectoral collaboration.

### 4.5 Implications for Policy and Practice

The findings of this study carry important implications for strengthening HIV care retention. At the policy level, potential strategies may include reinforcing ART supply chains, redesigning clinic infrastructure to safeguard both privacy and confidentiality, and institutionalizing interoperable digital systems for patient tracking.

In practice, providers could receive targeted training on confidentiality, while peer‐supported tracing and integrated psychosocial support could be expanded as routine components of care.

Community engagement is equally critical, requiring structured partnerships with faith‐based institutions and community leaders to reduce stigma and promote adherence. Finally, future research should evaluate the cost‐effectiveness of digital tracing tools, explore tailored interventions for pastoralist and mobile populations, and assess models of faith‐based collaboration to determine their impact on retention outcomes.

### 4.6 Strengths and Limitations

This study uses a qualitative, grounded theory approach to explore LTFU in HIV care, allowing findings to emerge organically from the data without prior assumptions. The study applied flexible interview guides based on literature and stakeholder input, a diverse participant group, and active stakeholder engagement to ensure practical relevance. However, group dynamics, such as dominant personalities, might affect the discussion during group interviews, where more vocal individuals may overshadow quieter participants.

## 5 Conclusions and Recommendations

This study identifies multifaceted drivers of LTFU in HIV care, emphasizing structural, social, and programmatic barriers that hinder patient retention; key findings include programmatic challenges such as drug supply interruptions and inadequate service integration, weak transfer, linkage, and tracing systems, social, cultural, and behavioral factors such as stigma and discrimination, and implementation gaps in SBC strategies. Addressing these issues may benefit from structural reforms, improved tracing and linkage systems, culturally sensitive interventions, and enhanced stakeholder engagement to support retention in HIV care and improve health outcomes for people living with HIV in the region.

## Author Contributions

**Fikru Tafese:** conceptualization, writing – original draft, writing – review and editing, investigation. **Matebu Gezahegn:** investigation, writing – review and editing, conceptualization, formal analysis. **Zeriun Kura:** writing – review and editing, writing – original draft. **Eshetu Alemayehu:** writing – review and editing, writing – original draft. **Yadeta Ayana:** validation, writing – review and editing. **Chala Bafikadu:** writing – review and editing, investigation, writing – original draft. **Gusha Belako:** supervision, writing – review and editing, investigation. **Tesfaye Zerfu:** supervision, writing – review and editing, investigation. **Birhanu Kenate Sori:** writing – review and editing, resources, investigation. **Yadeta Dassie:** supervision, resources, writing – review and editing, investigation. **Firaol Bekele:** investigation, writing – review and editing, conceptualization. **Negash Sime:** resources, conceptualization, methodology, writing – review and editing. **Tizta Tilahun Degfie:** conceptualization, writing – review and editing, investigation. **Daniel Yilma:** writing – review and editing, conceptualization, investigation. **Yohannes Ejigu:** writing – review and editing, supervision. **Yohannes Kebede:** conceptualization, writing – review and editing, formal analysis, supervision. **Mirkuzie Woldie:** supervision, writing – review and editing.

## Ethics Statement

Ethical approval for this study was obtained from the Institutional Review Board of Jimma University, Institute of Health (approval number: ref. no: JUIH/IRB/0521/25). The study was conducted in accordance with the ethical principles outlined in the Declaration of Helsinki. All participants were informed about the purpose of the study, assured of confidentiality, and provided verbal informed consent prior to participation. No personal identifiers were collected, and privacy was maintained during all interviews and discussions. All audio recordings and related data were stored securely on a password‐protected device accessible only to the principal investigator. Participation was voluntary, and individuals could withdraw at any time without consequence.

## Conflicts of Interest

The authors declare no conflicts of interest.

## Transparency Statement

The corresponding author affirms that this manuscript is an honest, accurate, and transparent account of the study being reported; that no important aspects of the study have been omitted; and that any discrepancies from the study as planned have been explained.

## Data Availability

The qualitative interview data generated and analyzed during this study are not publicly available due to confidentiality agreements with participants. De‐identified excerpts supporting the findings are available from the corresponding author upon reasonable request.
